# SafeHANDS: A Multimodal Hand Hygiene Intervention in a Resource-Limited Neonatal Unit

**DOI:** 10.3390/tropicalmed8010027

**Published:** 2022-12-29

**Authors:** Angela Dramowski, Louisa M. Erasmus, Marina Aucamp, Aaqilah Fataar, Mark F. Cotton, Susan E. Coffin, Adrie Bekker, Andrew C. Whitelaw

**Affiliations:** 1 Department of Paediatrics and Child Health, Faculty of Medicine and Health Sciences, Stellenbosch University, Cape Town 7500, South Africa; 2 Infection Control Service, Mowbray Maternity Hospital, Cape Town 7705, South Africa; 3 Division of Infectious Diseases, Children’s Hospital of Philadelphia, Philadelphia, PA 19104, USA; 4 Department of Paediatrics, University of Pennsylvania Perelman School of Medicine, Philadelphia, PA 19104, USA; 5 Division of Medical Microbiology, Department of Pathology, Faculty of Medicine and Health Sciences, Stellenbosch University and National Health Laboratory Service, Tygerberg Hospital, Cape Town 7505, South Africa

**Keywords:** hand hygiene, neonate, healthcare-associated infection, infection prevention, alcohol-based handrub, multi-modal intervention

## Abstract

Background: Hand hygiene (HH) is a cornerstone of programmes to prevent healthcare associated infections (HAI) globally, but HH interventions are seldom reported from African neonatal units. Methods: We conducted a quasi-experimental study evaluating the impact of a multi-modal intervention (SafeHANDS) on HH compliance rates, alcohol-based handrub (ABHR) usage, the Hand Hygiene Self-Assessment Framework (HHSAF) score, and healthcare-associated bloodstream infection (HA-BSI) rates at a 132-bed South African neonatal unit (4 wards and 1 neonatal intensive care unit [NICU]). The intervention included a campaign logo, HH training, maternal education leaflets, ABHR bottles for staff, and the setting of HH performance targets with feedback. Three 5-month study phases were completed in July 2020 (baseline), December 2020 (early) and May 2021 (intensive). Results: A total of 2430 HH opportunities were observed: 1002 (41.3%) at baseline, 630 (25.9%) at early and 798 (32.8%) at intensive study phases. At baseline, the overall neonatal unit HH compliance rate was 61.6%, ABHR use was 70 mL/patient day, and the baseline HHSAF score was ‘basic’ (165). The overall neonatal unit HH compliance rate was unchanged from baseline to intensive phases (617/1002 [61.6%] vs. 497/798 [62.3%]; *p* = 0.797). The ABHR use remained similar between phases (70 versus 73 mL/patient day). The HHSAF score improved to ‘intermediate’ level (262). There was no change in the neonatal unit HA-BSI rate. Conclusion: Despite improvement in the HHSAF score, no improvement in overall HH compliance rates, ABHR usage, or HA-BSI rates was observed. Future HH interventions in resource-limited neonatal units should incorporate implementation science and behaviour modification strategies to better understand the barriers and facilitators of HH best practice.

## 1. Introduction

Healthcare associated infection (HAI) remains a major threat to patient safety and a strain on healthcare resources globally [1,2]. Although data on the HAI burden in low- and middle-income country (LMIC) neonatal units is scarce, the risk of developing HAI may be up to 20-fold higher than in high-income countries [3]. The prevention of HAI in neonatal units is particularly challenging given the vulnerability of preterm and sick neonates, prolonged length of stay, use of invasive devices, antibiotic exposure and repeated handling for feeding, nappy changes, and observations. In LMIC neonatal units, neonatal infection risk is further increased by overcrowding, understaffing, lack of access to safe water supplies, and limited isolation facilities [4].

Most bacterial pathogens causing HAI are spread through direct contact, with healthcare workers’ hands being the most important vehicle. As many as 50–70% of HAI episodes are attributed to poor hand hygiene (HH) [5,6]. Consequently, ensuring high HH compliance is a cornerstone of HAI prevention to disrupt pathogen transmission, colonization, and infection [6,7,8,9,10]. In a systematic review including 96 studies (all from high-income countries), the median HH compliance rate among hospital-based healthcare workers was only 40%, even in intensive care units (ICU) [11]. Even lower compliance rates are reported from LMIC, including Tunisia (32%), Algeria (19%), Morocco (17%) [12], and India (12%).

Programs to improve HH compliance are often implemented within a multi-modal infection prevention intervention or care bundle [13]. The World Health Organization (WHO) launched multimodal HH campaigns in 2005 and later introduced the ‘5 Moments for Hand Hygiene’ [14,15] with an implementation strategy for different healthcare settings [8,16,17,18,19,20,21,22,23]. Two systematic reviews of HH interventions showed improved compliance rates (odds ratio 1.82, 95%CI 1.69–1.97) and a significant reduction in HAI (odds ratio 1.35; 95% CI 1.04–1.76), respectively [24,25]. Most HH interventions in hospitals target nursing personnel who provide the majority of direct patient care [5]. More recently, interventions have broadened to include other important role-players including multi-disciplinary healthcare staff and ‘lay caregivers’, such as mothers, in neonatal units [5,26]. In many LMIC settings, including India and South Africa, patients’ family members provide inpatient care and are crucial to the success of hospital-based HH interventions [27].

Despite efforts to improve HH measures, compliance is suboptimal in many LMIC neonatal units [11], with major gaps in healthcare workers’ HH knowledge and practices and scant evidence from African neonatal units. We evaluated the impact of a multi-modal intervention (SafeHANDS) on HH compliance rates, alcohol-based handrub (ABHR) usage, the Hand Hygiene Self-Assessment Framework (HHSAF) score [28], and healthcare-associated bloodstream infection rates at a large South African neonatal unit. 

## 2. Methods

### 2.1. Study Design, Population, and Setting

We conducted a quasi-experimental observation study using an uncontrolled before-and-after design to evaluate the impact of a multi-modal intervention (SafeHANDS) on HH compliance rates, ABHR use, the HHSAF score, and HA-BSI rates at a large neonatal unit in Cape Town, South Africa. Tygerberg Hospital is a 1384-bed public teaching hospital with a large obstetric-neonatal service delivering 8000 high-risk pregnancies and admitting 3000 neonates per year, 37% of whom are of low birthweight (<2500 g). The neonatal unit is the second largest in South Africa with 132 beds: a 12-bed neonatal ICU, three 30-bed high-dependency wards, and a kangaroo mother care (KMC) ward. The neonatal unit provides medical and surgical care for sick, preterm (<37 weeks’ gestation), and/or low birthweight inborn and outborn neonates. Prematurity, perinatal asphyxia and infection are common indications for admission. Each neonatal 30-bed ward is staffed by 2 registered nurses, and 4–5 staff grade nurses or enrolled nursing assistants per shift. During working hours, each ward has 1 consultant neonatologist, 1 neonatal fellow, and 3–4 junior doctors, including paediatric registrars, medical officers, and interns; at night and on weekends, only 2 junior doctors and one consultant are on duty for the 4 wards, with one consultant and one paediatric registrar on call in the NICU. Each ward has one household aid/cleaner on duty in the daytime, with allied healthcare workers covering multiple neonatal wards as required. In the NICU, most patients are ventilated, whereas in the wards (excluding the KMC ward), about 40% of all neonates receive high care, including non-invasive ventilation, surfactant administration, central lines, and total parenteral nutrition as needed.

### 2.2. Hand Hygiene Practices in the Neonatal Unit Prior to the SafeHANDS Intervention

The hospital’s infection prevention and control (IPC) program includes a major focus on HH with implementation of the WHO guidelines [14,15]. Following several large infection outbreaks, the neonatal unit made system changes to strengthen IPC [29], including the installation of wall-mounted ABHR dispensers at point-of-care (2013) and removing handwash basins from clinical rooms (2020). Each ward has handwash basins at the entrance with a continuous piped supply of clean water, hand soap, and disposable hand towels and automated ABHR dispensers at every cot. HH training for staff is performed during in-service training sessions on IPC throughout the year, during the WHO global HH awareness week annually, and on hospital admission for neonates’ parents. Ongoing audits of HH compliance are conducted at least every 6-months by the neonatal-obstetric IPC nurse practitioner and ward managers. Workplace reminders (HH posters) are displayed at strategic positions in the wards. The hospital’s IPC program (including HH modules) has institutional support from a committee comprising senior hospital medical, nursing, laboratory, and IPC leaders. This entails overt endorsement of the importance of hand hygiene, prioritizing the allocation of funds for infrastructural changes related to hand hygiene and hand hygiene supplies, expediting maintenance work required for hand washbasins, and prioritizing issues related to stock shortages of hand hygiene supplies. 

### 2.3. The SafeHANDS Multimodal Intervention

The SafeHANDS study was conducted over 15 months in three 5-month phases: baseline (July–November 2020), early (December 2020–April 2021) and intensive intervention (May 2021–September 2021). During the study period, peak COVID-19 transmission waves in the Western Cape Province occurred in July 2020, January 2021, and July 2021 [30]. The baseline phase recorded data on HH compliance rates, ABHR usage, and HHSAF score without implementing any interventions. In the early phase, study interventions included face-to-face training of staff on HH best practice incorporating videos using neonatal unit staff demonstrating HH best practice, and providing personal, refillable ABHR bottles to all staff. In the intensive phase, study interventions included designing the SafeHANDS logo, maternal infection prevention education leaflets, staff HH performance feedback using posters, in-person awareness sessions during global HH campaign week, launch of a HH commitment wall, provision of personal ABHR bottles, and the setting of compliance performance targets (Figure S1). Certificates (bronze, silver, and gold) were awarded to each ward at the hospital’s annual HH week celebrations in May 2022, based on the HH compliance rate during the SafeHANDS intervention program.

### 2.4. Education and Training on Hand Hygiene during the SafeHANDS Intervention

Neonate’s mothers received a brief educational demonstration about HH at neonatal unit admission and were provided with the IPC education leaflet, which included key messages about the importance of HH during the early and intensive phases. For mothers who could not read (<10%), the information leaflet’s key messages were conveyed using infographics and verbal explanations. To modify staff HH behaviour and compliance, training sessions were designed to impart knowledge and persuade neonatal staff that better HH practices would contribute to preventing neonatal infections. The HH training sessions at the start of the early study phase covered the following topics: the role of HH as a core IPC measure; the significance of HH in the “patient zone”; practical considerations for performing HH using the WHO 5 Moments for Hand Hygiene; revision of HH methods highlighting crucial steps in the hand washing/hand rubbing procedure with return demonstrations by staff; the mother/caregiver’s HH role in the neonatal wards; and how the hand wash station functioned at each ward entrance. Different case scenarios were presented for discussion using video material (e.g., *Should hand hygiene be performed before or after opening the portholes of the incubator? Does the wearing of gloves negate the need for hand hygiene?).* To ensure that the staff could relate to the content of the training, a set of short videos was made within the hospital’s neonatal wards with neonatal staff acting out the different scenarios aiming to improve HH knowledge and attitudes.

### 2.5. Data Sources and Outcomes of Interest

Four study endpoints were measured during the baseline, early, and intensive intervention phases:

1. HH compliance rates (by ward and for the neonatal unit overall): for each of the 5 neonatal wards, including NICU, a minimum of 150 direct HH observations were conducted in each study phase by 3 discrete trained observers using the WHO HH observation tool [31] converted to a RedCAP form for mobile devices [32]. HH compliance rates were reported as percentages for each ward and for the neonatal unit overall;
2. ABHR usage (by ward and for the neonatal unit overall): the volume of ABHR used by each neonatal ward during each study phase was obtained from the hospital pharmacy dispensing records. To account for ward size and bed occupancy, the volume of ABHR used was divided by the patient days per ward per study phase × 1000. ABHR usage (for each ward and the neonatal unit overall) was reported as the total volume used in litres, as millilitres used per patient day, and as estimated HH actions/patient day (assuming an average of 3 mL ABHR used per opportunity);
3. The WHO HH self-assessment framework (HHSAF) score (for the neonatal unit overall) [33,34]: this self-administered validated questionnaire was performed at the study baseline (July 2020) and again following completion of the established study phase (October 2021) to systematically evaluate HH structures, resources, promotion, and practices at the facility. The HHSAF includes 27 indicators in 5 sections, corresponding to the core components of the WHO multimodal HH improvement strategy (system change, training and education, observation and feedback, reminders in the workplace, and hospital safety climate). Question responses were converted to numerical scores per component, producing an overall score sub-categorised into 4 levels of HH practice (inadequate, basic, intermediate, and advanced) [33];
4. Healthcare-associated bloodstream infection (HA-BSI) rate (for the neonatal unit overall): a laboratory-confirmed HA-BSI episode was defined as a blood culture collected > 72 h after unit admission with the isolation of a known pathogen. Organisms were classified using the United States Centers for Disease Control (CDC) list of pathogens and contaminants [35]. Repeat blood cultures isolating the same pathogen within 14 days of the original specimen were considered to represent a single episode of infection. Patients who isolated coagulase-negative staphylococci (CoNS) from two separate blood cultures taken 24–48 h apart were included as pathogens. All other contaminants were excluded. The HA-BSI rate was calculated for each study phase by dividing the total HA-BSI episodes by the total patient days for the neonatal unit in that 5-month period × 1000.

### 2.6. Statistical Analysis

The sample size required to deliver a 95% confidence interval with a 5% margin of error at an estimated HH compliance rate of 50% was calculated at a minimum of 384 HH observations per study phase. Continuous variables were reported as means and standard deviations if normally distributed and medians with interquartile ranges (IQR) if non-normally distributed. Categorical data was reported as proportions and percentages. Students t-tests, Pearson’s chi-square tests, and Fishers Exact tests were used for hypothesis testing as appropriate. For all statistical tests performed, a *p*-value < 0.05 was considered significant. All statistical analyses were performed using STATA 17.0 (College Station, Texas 77845, USA). The Stellenbosch University Health Research Ethics Committee and the Tygerberg Hospital management reviewed and approved the study protocol (N18/07/068).

## 3. Results

A total of 2430 HH opportunities were observed over the 15-month study period, 1002 (41.3%) at baseline, 630 (25.9%) at early, and 798 (32.8%) at intensive study phases. 

### 3.1. Baseline Observations

**Figure 1:**
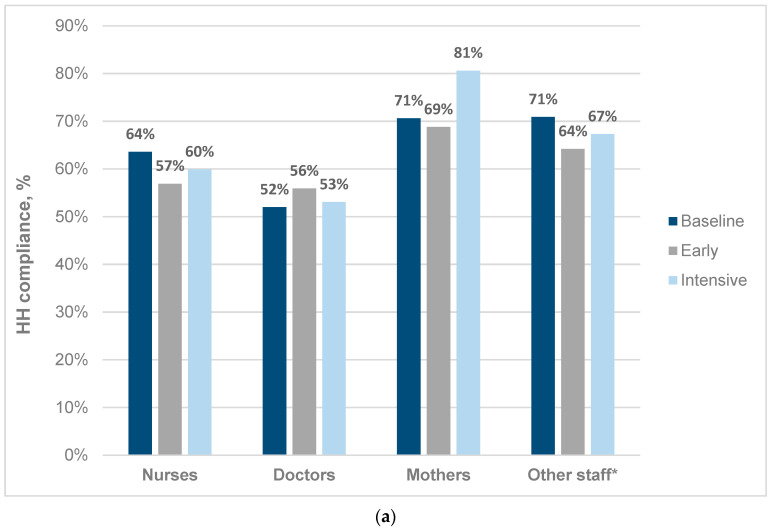

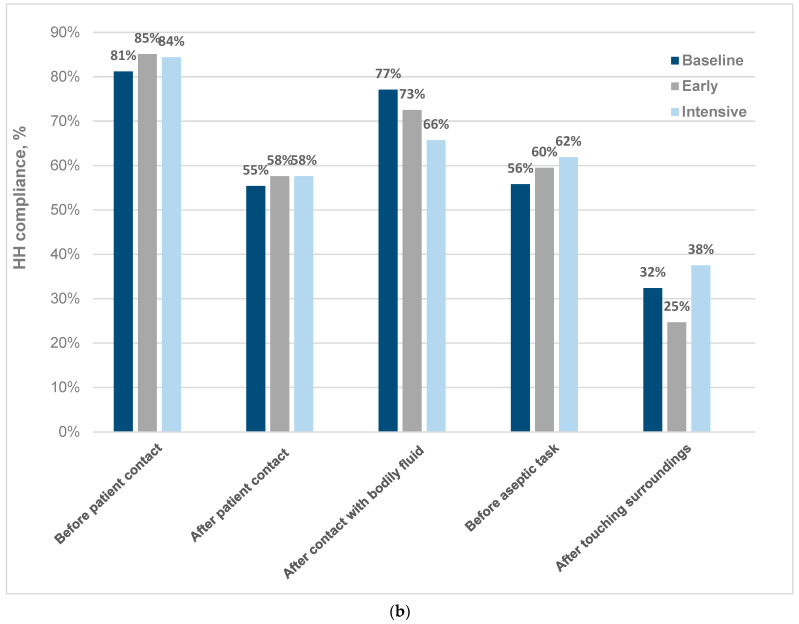
(**a**) Hand hygiene compliance rates by neonatal unit role and study phase. (**b**) Hand hygiene compliance by WHO five moments for HH type and study phase. * Other staff = cleaners, porters, radiology and allied health staff (physiotherapists, dieticians, porters, ward clerks, occupational and speech therapists). HH compliance = HH performed/HH moments observed × 100 (% compliance).

At baseline, the overall neonatal unit HH compliance rate was 61.6% (617/1002; 95% CI 58.5–64.5%). Baseline observed HH compliance rates were lower in the four neonatal wards than the NICU (489/820 [59.6%] versus 128/182 [70.3%]; *p* = 0.009) (Table 1). Baseline HH compliance rates were highest in ‘other staff’ including radiographers, dieticians, porters, ward clerks (39/55; 70.9%) and mothers (108/153; 70.6%), and lowest in doctors (157/302; 52.0%) and nurses (313/492; 63.6%) (Figure 1a). For all roleplayers, the highest baseline HH compliance rates were recorded before patient contact (81.2%) and after contact with bodily fluids (77.1%), with the lowest HH compliance rates after touching patient surroundings (32.4%) (Figure 1b). ABHR was used for hand decontamination much more frequently than hand washing; 554/617 (89.7%) HH opportunities were observed using ABHR vs. 63/617 (10.3%) using handwashing at baseline. The baseline volume of ABHR use was 70 mL/patient day equating to an estimated 23 HH actions per patient per day (Table 2). The WHO HHSAF score prior to the implementation of intervention was 165, ranking the neonatal unit’s HH practices at a ‘basic’ level, with the lowest scoring baseline categories being ‘education and training’, ‘workplace reminders’, and ‘institutional safety climate’ (Table 3). During the baseline 5-month period, neonatal unit occupancy was 85%, with 2.9 HA-BSI episodes/1000 patient days. In-person neonatal unit HH training was conducted at the end of the baseline period with 141 staff (55 doctors and 86 nurses), representing approximately 60% of total neonatal unit staff, both day and night shifts.

### 3.2. Changes after the SafeHANDS Intervention

The overall neonatal unit HH compliance rate was unchanged from baseline to intensive phases (617/1002 [61.6%] vs. 497/798 [62.3%]; *p* = 0.797) (Table 1). The only improvement in HH compliance rate by the neonatal unit role was observed in mothers, although the absolute increase was not statistically significant (108/153 [70.6%] vs. 58/72 [80.6%]; *p* = 0.171) (10% between the baseline and intensive intervention phase; 108/153 [70.6%] vs. 58/72 [80.6%]; *p* = 0.171) (Figure 1a). The volume of ABHR used remained similar between phases at 70 versus 73 mL/patient day (Table 2). The volume of ABHR used during the SafeHANDS intervention correlated with improvements or decline in the HH compliance rates observed in neonatal wards 1 and 3, and the KMC ward respectively (Table 2). The estimated HH actions per patient day were low, ranging from 9 handrubs per day in the KMC ward to 27 in the NICU during the intensive phase (Table 3). Post-intervention, the neonatal unit’s HHSAF score improved to 262.5 (‘intermediate’ level), with the largest improvements achieved in the ‘workplace reminders’, ‘institutional safety climate’ categories and ‘education and training’ domains (Table 3). Neonatal bed occupancy rate was sustained at a high level throughout the study (85–91%). There was no change in the neonatal unit HA-BSI rate during the SafeHANDS intervention from 2.9 to 3.1 episodes/1000 patient days between baseline and intensive phases (Table 4). 

## 4. Discussion

This multimodal HH intervention program (SafeHANDS) in a large hospital neonatal unit demonstrated low-moderate HH compliance rates (55–70%) at baseline, with no significant changes in overall HH compliance post-intervention. Neonates’ mothers demonstrated the highest HH compliance rates overall and the greatest percentage increase in compliance post-intervention. The WHO HHSAF score improved from ‘basic’ to ‘intermediate’ level by increases in the ‘education and training’, ‘workplace reminders’, and ‘institutional safety climate’ domain scores. The volume of ABHR used did not change, but it correlated closely with HH compliance rates in each phase. Neonatal HA-BSI rates were unchanged during the 3-phase HH intervention.

The HH compliance rates observed at baseline (55–70%), early (52–63%), and intensive (44–69%) intervention phases of the SafeHANDS program were consistently higher than the 40% (range: 5–89%) reported among HCWs in other LMIC settings [15,36]. We observed the highest HH compliance rates in all study phases in the NICU, as has been documented internationally [10,37,38]. The higher rates of HH compliance observed in neonatal wards and NICUs may be attributable to healthcare workers’ perceptions of increased infection vulnerability in neonates.

Most published intervention studies from neonatal units report substantial HH compliance improvements, but the seldom report on impact on neonatal HA-BSI rates. A study from a Special Neonatal Care Unit in India demonstrated an improvement of 30% in mean HH compliance following the implementation of HH posters, HH educational sessions, provision of HH consumables, and weekly performance feedback and discussions [39]. A Ghanaian neonatal ICU achieved improved HH compliance from 67% to 92% compliance with training, practice reinforcement, visual reminders, and continuous provision of HH consumables [40]. In an Iranian NICU, HH compliance rates increased from 30% to 70% following the implementation of HH training and direct observation [41]. Similarly, an Indian NICU increased HH compliance rates from 46% to 69% following intensified HH training and staff education [42]. Despite implementation of the SafeHANDS intervention incorporating program elements shown to successfully increase HH compliance [24,25], we did not achieve overall improvement (61.6% vs. 62.3% compliance from baseline to intensive phases). However, one of the four acute care ward neonatal wards did achieve a 12% improvement in HH compliance. 

A likely reason for the lack of impact of the SafeHANDS program is a failure to achieve behavioural modification among staff (i.e., increased HH knowledge did not translate into improved HH practice). Although visual HH reminders were present in all neonatal wards, the lack of daily reminders and designated HH champions for each shift may have impeded our ability to impact behaviour. The development of HH champions (nurses, doctors, and mothers in each ward) as role models to proactively demonstrate correct HH practices, praise staff, and mothers for HH well-performed and to tackle non-compliance in a non-punitive manner, and they would assist in building an “HH culture.” We did not investigate neonatal unit staff beliefs, attitudes, and perceived behavioural control regarding HH prior to the SafeHANDS programme. Future interventions should use validated theoretical frameworks (e.g., the theory of planned behaviour model) [43] to inform developing the HH program [11]. Various other factors may have contributed to suboptimal HH compliance including logistical barriers such as high bed occupancy rates, understaffing, time constraints, and behavioural factors, such as skepticism about the value of HH and forgetfulness [15,44,45,46]. Full compliance may be an unrealistic goal, especially in neonatal settings where frequent patient handling is required, but high levels of HH compliance (i.e., >70–90%) are essential to reduce infection risk.

In keeping with global HH study findings, medical doctors had substantially lower observed HH compliance rates than other healthcare workers (nurses, cleaners, allied staff, etc.) in all study phases [37,47,48]. Perhaps unsurprisingly, neonates’ mothers had the highest observed HH compliance rates (72.6%) and demonstrated the greatest percentage increase in compliance rates (10%) overall. This is in keeping with findings from 4 South African hospitals, which showed overall neonatal unit HH compliance rates of 70.6%, 63.6%, and 52.2% in mothers, nurses, and doctors, respectively [49]. Given the critical role of nursing personnel and mothers in neonatal inpatient care, hospital HH programs in LMIC should specifically target these two key groups [27].

The HH compliance assessments using the WHO ‘5 Moments for Hand Hygiene’ are included in most IPC guidelines [14,15]. In keeping with two LMIC NICU HH studies [50,51], we observed the most frequently missed HH opportunities were following ‘contact with patient surroundings’ (34.8%) and ‘after touching the patient’ (56.7%). Healthcare workers may perceive these HH opportunities as less critically important for patient safety than HH prior to patient contact, for example. However, special attention to these 2 HH moments is needed, as they contribute heavily to bacterial contamination of the patient zone. Conversely, HH compliance following contact with bodily fluids was comparatively high (73.3%), possibly owing to healthcare workers’ desire to protect themselves from the risk of contamination [11].

Quantitative measurement of the volume of ABHR use is a useful, albeit inexact, proxy for HH compliance rates, especially in our setting where ABHR use was 10-fold higher than handwashing. We found that the ABHR volume used mirrored the trend in HH compliance rates observed in each ward in each study phase (unchanged, increased, or decreased). Although direct observed HH compliance monitoring is the gold standard, useful insights may be gained by tracking trends in ABHR usage. However, intermittent direct observation is essential to evaluate staff HH practices and identify opportunities for improvement [52].

In LMIC neonatal units where supplies of clean water and HH consumables may be interrupted, easy access to and use of the WHO-advocated ABHR should be promoted. The prior removal of handwash basins in 2020 following repeated outbreaks of Gram-negative sepsis has clearly influenced the move to ABHR as the dominant HH method in our neonatal unit, with the added benefits of timesaving and emollients for hand protection. However, in many LMIC, the continuous availability of ABHR remains a challenge, with only 17% availability reported compared to 75% in high-income countries [53].

The WHO recommends that healthcare facilities use the HHSAF annually to track institutional performance and progress in HH initiatives [34,54]. During the course of the SafeHANDS program, the neonatal unit’s HHSAF score improved by one level from ‘basic’ to ‘intermediate’, driven by improvements in the ‘workplace reminders’, ‘institutional safety climate’, and ‘education and training’ categories. To achieve further improvement, the neonatal unit (and indeed the whole 1384-bed institution) should allocate a dedicated budget for HH training, ensure mandatory staff HH training with knowledge assessments annually, implement regular HH audits with feedback, make WHO HH materials readily available, improve the use of workplace HH reminders, and develop HH role models and a HH patient engagement plan. Compared to other WHO regions, the Africa region has the lowest HHSAF scores (mean 281, SD 127), possibly owing to poor healthcare infrastructure, lack of infection prevention resources, and a limited knowledge of HH implementation and sustainability [55]. Our post-intervention neonatal unit HHSAF score of 262 (intermediate level) is in keeping with scores reported from other LMIC settings with median HHSAF survey scores ranging from 233 in low-income countries to 395 in high-income countries [53,56]. The disparity between the HHSAF and the HH compliance and other related variables highlights the fact that institutional progress and commitment to HH does not always achieve behavior change at the level of the individual. In our study, although the HHSAF score improved following HH education interventions and additional workplace reminders, we did not achieve HH compliance rate improvement in the neonatal unit.

Given the lack of improvement in HH compliance following the SafeHANDS intervention, it was unsurprising that there was also no change in the neonatal HA-BSI rate. High neonatal bed occupancy rates (85–91%) and suboptimal staff-to-patient ratios contributed to the challenges in ensuring compliance with HH recommendations and other infection best practices. This highlights the need to implement multimodal infection prevention strategies in LMIC neonatal units, where the causal pathway from colonisation to infection is particularly complex [56]. The study was not designed to explain differences in the effectiveness of the intervention in healthcare workers versus mothers. This is an important area for future research, which should include a qualitative research component to better understand attitudes, barriers and facilitators to optimal hand hygiene practice in neonatal healthcare settings.

The SafeHANDS programme and study had several limitations, including the single-centre intervention, the possibility of the Hawthorne effect (improved compliance while being observed), and the limitation of HH observation to daytime and weekday shifts only. As multiple interventions were carried out concurrently, it was not possible to assess the impact of any single intervention during the study. Obtaining more information on the effectiveness of intervention element would help to prioritise and inform future hand hygiene quality improvement programs. Strengths of the study are, however, the large number of HH observations and long study follow-up period and the inclusion of mothers as crucial roleplayers and partners in patient safety in LMIC neonatal units. 

## 5. Conclusions

Although globally acknowledged as a core component of IPC programmes, few LMIC neonatal units have achieved an advanced level of HH promotion and practice. The SafeHANDS programme evaluated the impact of a multi-modal intervention on HH compliance, ABHR usage, the HHSAF score, and HA-BSI rates at a large South African neonatal unit. Despite improvement in the HHSAF score, no improvement in HH compliance rates, ABHR usage, or HA-BSI rates was observed. Future intervention studies should involve the implementation science and behaviour modification methods to better understand the barriers and facilitators of HH best practice in LMIC neonatal units. 

## Figures and Tables

**Table 1:** Observed hand hygiene (HH) compliance rates by neonatal ward type and study phase.

Phase Ward	Baseline Phase HH Compliance N (%)	Early Phase HH Compliance N (%)	Intensive Phase HH Compliance N (%)	% Change in HH Compliance *	*p*-Value	HH Target Level Achieved ^#^
Overall unit	617/1002 (61.6%)	369/630 (58.6%)	497/798 (62.3%)	+0.7%	0.797	silver
Neonatal intensive care unit	128/182 (70.3%)	67/106 (63.2%)	157/227 (69.2%)	−1.1%	0.883	silver
Ward 1	118/212 (55.7%)	66/127 (52%)	105/155 (67.7%)	+12.0%	0.025	silver
Ward 2	142/206 (68.9%)	87/143 (60.8%)	81/134 (60.4%)	−8.5%	0.135	silver
Ward 3	116/201 (57.7%)	108/178 (60.7%)	94/144 (65.3%)	+7.6%	0.190	silver
Kangaroo Mother care ward	113/201 (56.2%)	41/76 (53.9%)	60/136 (44.1%)	−12.1%	0.038	bronze

* measured change in HH compliance rate between baseline and intensive phases; HH compliance = HH performed/HH moments observed × 100 (% compliance). ^#^ Performance targets for HH compliance rates were set as follows: bronze = 50–59%; silver = 60–69%; gold = 70% or more.

**Table 2:** Changes in alcohol-based handrub usage during the SafeHANDS intervention.

Intervention Phase	Baseline	Early	Intensive	Change in Volume of ABHR Used # (Percentage)
**Overall neonatal unit** ABHR volume used (litres) ABHR used (mL/patient day) Estimated HH actions/patient day *	1203.5 70 23	1111 61 20.3	1337.5 73 24	+11%
**Ward 1** ABHR volume used (litres) ABHR used (mL/patient day) Estimated HH actions/patient day *	301 67 22	276 61 20	301 67 22	0%
**Ward 2** ABHR volume used (litres) ABHR used (mL/patient day) Estimated HH actions/patient day *	283.5 63 21	261 68 19	376 84 28	+33%
**Ward 3** ABHR volume used (litres) ABHR used (mL/patient day) Estimated HH actions/patient day *	281 62 21	341 76 25	396 88 29	+42%
**Kangaroo Mother care ward** ABHR volume used (litres) ABHR used (mL/patient day) Estimated HH actions/patient day *	188 42 14	128 28 9	118 26 9	−38%
**Neonatal ICU** ABHR volume used (litres) ABHR used (mL/patient day) Estimated HH actions/patient day *	150 83 27	105 58 19	146.5 81 27	−2.4%

ABHR = alcohol-based handrub; HH = hand hygiene; mL = milliliters; * Estimated number of hand hygiene actions performed for each occupied bed assuming an average of 3 mL handrub used; # percentage change in ABHR volume used from baseline to intensive phase.

**Table 3:** Hand hygiene self-assessment framework scores before and after implementing the SafeHANDS intervention.

Component	Pre-Implementation (November 2020)	Post-Implementation (October 2021)
1. System change	75/100	75/100
2. Educational and training	15/100	40/100
3. Evaluation and feedback	40/100	45/100
4. Reminders in the workplace	20/100	52.5/100
5. Institutional Safety Climate	15/100	50/100
**Total score**	165 **(Basic level)**	262.5 **(Intermediate level)**

**Table 4:** Neonatal unit healthcare-associated bloodstream infection rate.

Metric	Baseline	Early	Intensive
Neonatal unit bed occupancy per study phase	85.6%	90.5%	91.0%
Neonatal unit HA-BSI episodes	49	66	56
Neonatal unit patient days per study phase	17,239	18,218	18,314
Neonatal HA-BSI rate per 1000 patient days	2.9	3.6	3.1

HA-BSI = healthcare-associated bloodstream infection rate.

## Data Availability

The datasets generated during and/or analysed during the current study are available from the corresponding author on reasonable request.

## Supplementary Materials

The following supporting information can be downloaded at: https://www.mdpi.com/article/10.3390/tropicalmed8010027/s1, Figure S1: SafeHANDS training interventions for neonatal staff.

## Abbreviations

ABHR	ABHR
HH	Hand hygiene
HHSAF	HH self-assessment framework
IPC	Infection Prevention and Control
LMIC	Low- and Middle-Income Countries
WHO	World Health Organization
