# Supplementary material for: SafeHANDS: A Multimodal Hand Hygiene Intervention in a Resource-Limited Neonatal Unit
Source: Trop Med Infect Dis. 2022 Dec 29;8(1):27. doi: 10.3390/tropicalmed8010027 (PMC9867086; doi:10.3390/tropicalmed8010027)
Supplement: Supplementary file 1 [file tropicalmed-08-00027-s001.zip › tropicalmed-1885365-supplementary.pdf]

Tropical Medicine and Infectious Disease

MDPI

Supplementary Materials for SafeHANDS: a Multimodal Hand Hygiene Intervention in a Resource-Limited Neonatal Unit

Figure S1. SafeHANDS training interventions for neonatal staff.

Staff hand hygiene training sessions, incorporating other key infection prevention elements

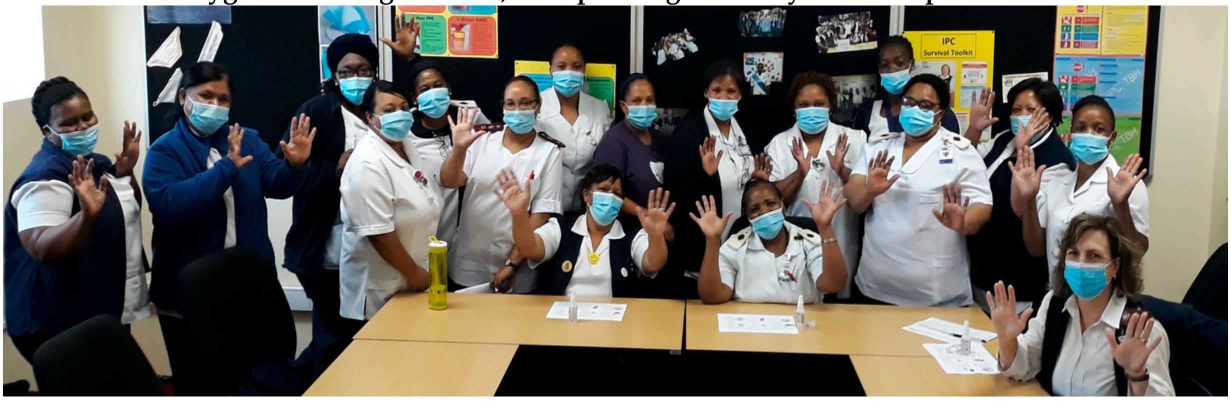

Creation of a hand hygiene training video featuring neonatal unit staff members

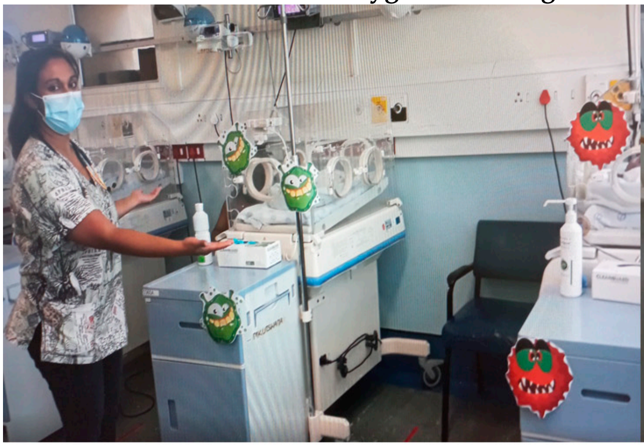

The patient zone

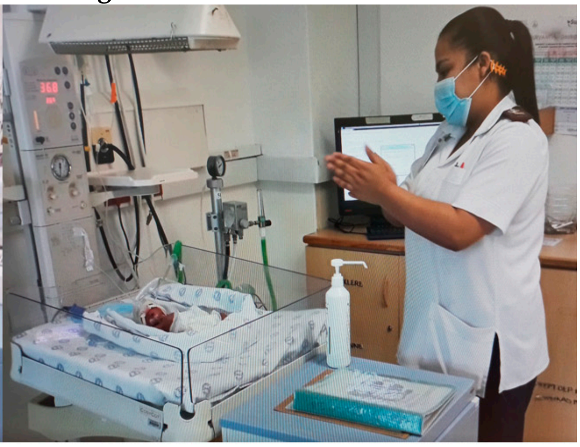

HH moment '1'

Creation of posters for hand hygiene audit feedback and the SafeHANDS campaign logo

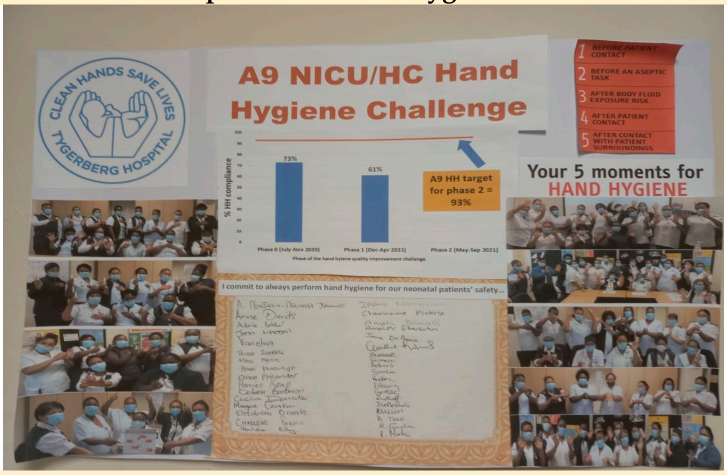

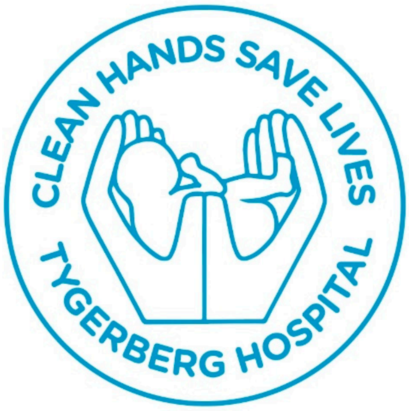

Signing of the Hand Hygiene Commitment wall during the global HH week

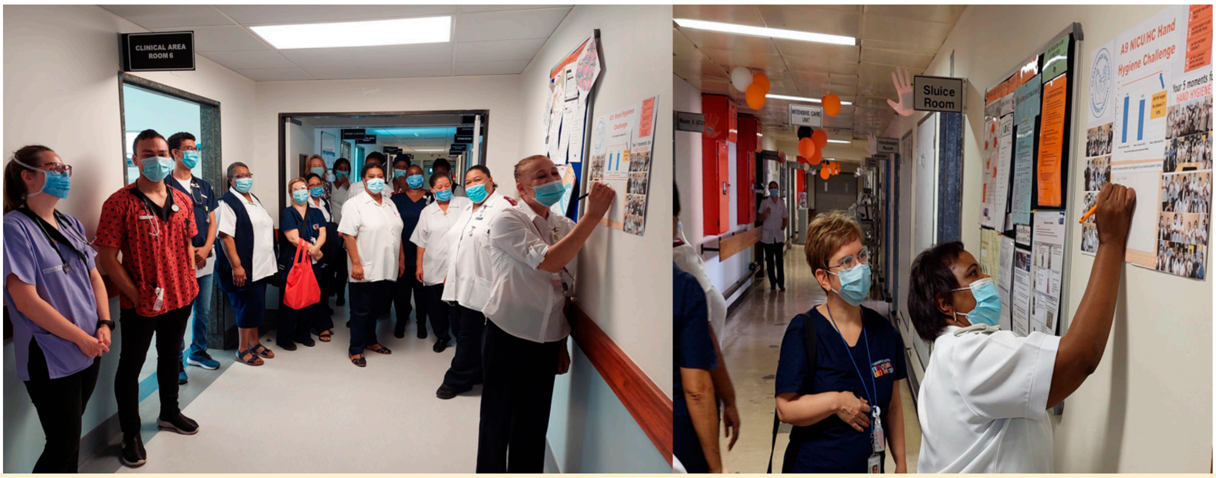

Development of an infection prevention education leaflets for parents

Your baby's safety is important to us

Dear Parent, the health and safety of you and your baby are important to us. There are many ways that you can help to keep your baby free from infection during their hospital stay. *Thank you! Let's work together for patient safety!* Tygerberg Hospital Staff

**Clean your hands**<br>Wash your hands when:<br>- you enter the ward<br>- before touching baby<br>- after touching baby<br>- after changing the nappy<br>- after going to the toilet<br>- after touching your phone.	**Practice good personal hygiene**<br>The bacteria on your body and clothes may be transferred to, and cause infection in your baby.<br>Whenever possible please try to wear clean clothes and wash your body daily.	NO CELLPHONES<br>**Don't touch your phone**<br>Cellphones carry many harmful bacteria. Touching your phone and then your baby or surfaces in the ward can spread dangerous bacteria.
**Don't touch other babies**<br>Do not touch any babies, surfaces or equipment as this can spread germs. Even if you want to help the other children, you may pick up their germs and carry the germs back to your child.		
**Clean the surfaces**<br>Wipe down the incubator, cot and bedside cabinet daily with a disinfectant cloth.<br>The nursing staff will show you where to find the cloths and how to use them.	**Report symptoms immediately**<br>If you are feeling sick with fever, cough, muscle aches etc please tell the nurse or doctor before entering the ward.<br>Germs from adults can cause severe illness in babies.	**No food**<br>Do not bring any food into your baby's room.<br>Please eat and store all food items in the ward kitchen. The ward staff will show you how to label your food and store it in the kitchen refrigerator.
